# Effects of a Lutein and Zeaxanthin Intervention on Cognitive Function: A Randomized, Double-Masked, Placebo-Controlled Trial of Younger Healthy Adults

**DOI:** 10.3390/nu9111246

**Published:** 2017-11-14

**Authors:** Lisa M. Renzi-Hammond, Emily R. Bovier, Laura M. Fletcher, L. Stephen Miller, Catherine M. Mewborn, Cutter A. Lindbergh, Jeffrey H. Baxter, Billy R. Hammond

**Affiliations:** 1 Department of Psychology, The University of Georgia, Athens, GA 30602-3013, USA; lrenzi@uga.edu (L.M.R.-H.); emily.bovier@oswego.edu (E.R.B.); fletcher.lauram@gmail.com (L.M.F.); lsmiller@uga.edu (L.S.M.); mewborn@uga.edu (C.M.M.); cal@uga.edu (C.A.L.); 2 Institute of Gerontology, Department of Health Promotion and Behavior, College of Public Health, The University of Georgia, Athens, GA 30602, USA; 3 Department of Psychology, State University of New York, Oswego Campus, Oswego, NY 13126, USA; 4 Abbott Nutrition, Global Research and Development, Columbus, OH 43219, USA; jeffrey.baxter@abbott.com

**Keywords:** xanthophylls, cognition, reasoning, visual memory, attention

## Abstract

**Background:** Past studies have suggested that higher lutein (L) and zeaxanthin (Z) levels in serum and in the central nervous system (as quantified by measuring macular pigment optical density, MPOD) are related to improved cognitive function in older adults. Very few studies have addressed the issue of xanthophylls and cognitive function in younger adults, and no controlled trials have been conducted to date to determine whether or not supplementation with L + Z can change cognitive function in this population. **Objective:** The purpose of this study was to determine whether or not supplementation with L + Z could improve cognitive function in young (age 18–30), healthy adults. **Design:** A randomized, double-masked, placebo-controlled trial design was used. Fifty-one young, healthy subjects were recruited as part of a larger study on xanthophylls and cognitive function. Subjects were randomized into active supplement (*n* = 37) and placebo groups (*n* = 14). MPOD was measured psychophysically using customized heterochromatic flicker photometry. Cognitive function was measured using the CNS Vital Signs testing platform. MPOD and cognitive function were measured every four months for a full year of supplementation. **Results:** Supplementation increased MPOD significantly over the course of the year, vs. placebo (*p* < 0.001). Daily supplementation with L + Z and increases in MPOD resulted in significant improvements in spatial memory (*p* < 0.04), reasoning ability (*p* < 0.05) and complex attention (*p* < 0.04), above and beyond improvements due to practice effects. **Conclusions:** Supplementation with L + Z improves CNS xanthophyll levels and cognitive function in young, healthy adults. Magnitudes of effects are similar to previous work reporting correlations between MPOD and cognition in other populations.

## 1. Introduction

A wide body of research, across disciplines, has shown a direct effect of diet on nearly every aspect of brain function [1]. One big category of effect appears to be simply prophylactic. The brain is a strongly lipid-based, highly oxygenated structure prone to inflammatory stress. Dietary intake, good or bad, can influence the oxidative and inflammatory state of the brain and, hence, its function [2]. Another category is acute effects on cellular metabolism [3]. Dietary intake is involved in every aspect of a neuron’s function, ranging from influencing the basic structure of the cell itself (e.g., phospholipid composition [4]); to resting and action potentials; to neurotransmitter synthesis from amino acid and vitamin precursors (e.g., pyridoxal 5′-phosphate, a form of Vitamin B_6_, is a coenzyme required for the biosynthesis of gamma-aminobutyric acid (GABA), norepinephrine and serotonin from glutamic acid, tyrosine and tryptophan, respectively). B_6_, B_12_ and folate are required to support methylation reactions, some of which are noted above, and are also important in gene regulation. It is interesting to note that methylation is the sole method for regulation of genes in mitochondrial DNA, and that these vitamins are critical for mitochondrial function.

Like many areas of nutrition, most research on the diet–brain connection has focused on micronutrient deficiencies, such as the role of B vitamins in cognitive decline or diseases such as Korsakoff’s and dementia [5,6]. Much less is known about the many other phytochemicals that are commonly found in food (i.e., not yet defined as essential or even as nutrients). Even less is known about how those components of diet influence the brains of young healthy adults. One exception to this general trend is a growing interest in a possible role for the xanthophylls, specifically lutein (L), zeaxanthin (Z) and meso-zeaxanthin (MZ) [7]. These dietary carotenoids concentrate in neural tissue (particularly, the macula or central retina, there termed macular pigment (MP)). Unlike many dietary components, the amount of retinal L, Z and MZ can be measured directly and non-invasively [8]. MPOD has been shown to be highly correlated to L + Z accumulation in brain [9]. This allows the concentration of these xanthophylls in neural tissue to be correlated with dependent measures of interest. Using this technique, a number of cross-sectional studies have correlated the macular xanthophylls with many aspects of both visual and cognitive function in both young and older subjects [10,11,12,13,14,15].

The ability to directly measure the macular pigments also provides the additional advantage of being able to monitor the results of an intervention; to wit, one can supplement L, Z or MZ, measure changes in MP, and then assess any possible functional changes that result (and how those changes relate to the actual change in tissue levels). Using this strategy, a number of studies have now shown that supplementing L and Z is related to both increases in the amount of L + Z measured in the retina and alteration in behaviors that are thought to be primarily mediated by the brain. For example, supplementation with carotenoid-rich avocados causes increases in both the macular pigments and spatial working memory [16]. This improvement was not found for control subjects whose carotenoid levels did not increase. L + Z supplementation in healthy older adults produces improvements in cognitive function on a variety of domains [17,18,19]. Such effects have even been found in young healthy subjects (i.e., those seemingly less likely to change). For example, Bovier et al. [14,15] supplemented young subjects and found changes in visual temporal processing speed in subjects whose MP increased by 0.1 log units of optical density or more, versus those that did not, using a placebo-controlled design. Visual processing speed was chosen because, although it is a lower level visual function (basically, the fusion of square-and sine wave modulated light), it is thought to be mediated by central brain mechanisms (e.g., [20]). Indeed, some have argued that processing speed in general underlies many more complex cognitive traits [21] and should be considered a cognitive fundamental [22]. The results by Bovier et al. implied the possibility that supplementing xanthophylls could change other aspects of cognition (such as memory) even in young healthy subjects at the (apparent) peak of their cognitive life (e.g., college students). The present study was a first test of this hypothesis.

## 2. Materials and Methods

### 2.1. Subjects

This sub-study was part of a larger clinical trial on xanthophyll supplementation and cognitive function. For the young adult sub-study, a total of 79 potential participants (college students from the Athens-Clarke County, GA area) were screened for enrollment between August 2012 and December 2013, with follow-up lasting through December 2014. Of those 79 potential participants, three participants were initially excluded for failure to meet the inclusion criteria. Randomization to either the active supplement group or the placebo group was accomplished as follows: a set of numerical codes was created that corresponded to either the active supplement or the intervention. The codes were placed into an opaque envelope. After informed consent was signed and inclusion/exclusion criteria were verified (see below), a code was drawn from the envelope by the clinical coordinator, who had no data collection responsibilities. From the 76 participants who were deemed eligible for participation, 30 participants were randomized into the placebo group, and 46 total participants were randomized into the intervention group. Of those participants, two members of the placebo group and three members of the intervention group withdrew from the trial due to scheduling conflicts prior to completion of baseline measurements and allocation of supplements. Given the fact that students enrolled in the trial occasionally graduated and moved away during the study, 14 total placebo participants and 9 total intervention participants were lost to follow-up during the course of the year-long intervention.

The final analyzable data set included 51 healthy subjects ranging in age from 18 to 30 (*M* = 21.21 ± 2.52) years. Approximately equal numbers of males and females (*n* = 29 males, 22 females) were analyzed, with a total of *n* = 14 participants in the placebo group, and *n* = 37 participants who received the intervention. The active supplement contained 10 mg L + 2 mg Z (DSM Nutritional Products; Kaiseraugst, Switzerland), and the placebo was visually identical to the active supplement. Supplement and placebo containers were visually identical, with the exception of the numerical code on the bottle contents label. Participants were instructed to take one tablet per day with their highest fat meal. Compliance with the supplement regime was monitored by bi-monthly phone calls as well as pill counts.

The baseline characteristics of the entire analyzable young adult sample, as well as the two sub-groups, are shown in Table 1. The majority of the subjects were students from the University of Georgia, and placebo and active supplement groups were not significantly different from each other in demographic characteristics. Inclusion criteria included good overall and ocular/refractive (20:40 best corrected acuity) health and no supplement use in the previous six months (excluding a multivitamin containing less than 1 mg LZ/day).

### 2.2. Ethics

The tenets of the Declaration of Helsinki were adhered to at all times during the course of this study. All participants both verbally consented to participation and provided written informed consent prior to participation. The protocol was approved by the University of Georgia Institutional Review Board (2012105172).

### 2.3. Methods

#### 2.3.1. Retinal L + Z Levels

Retinal L + Z levels (as macular pigment optical density, MPOD) was measured at 30-min of retinal eccentricity along the horizontal meridian of the temporal retina, using customized heterochromatic flicker photometry (cHFP) [23,24]. Briefly, a macular densitometer (Macular Metrics; Rehoboth, MA, USA) was used to present participants with a one-degree test stimulus composed of light generated by two narrow-band LEDs, peaking at 460 nm (strongly absorbed by MP) and 570 nm (not absorbed by MP), respectively. Each waveband was presented in counter-phase orientation at two different starting intensities, which, when combined, appeared to participants as a single, uniform, flickering disk. The stimulus was presented on a 470 nm background.

Participants were required to centrally view the stimulus, and to adjust the intensity of the 460 nm test light relative to the 570 nm reference light until the appearance of flicker was minimized. The participants then viewed a larger (two-degree) stimulus while fixating on a point at 7-degrees of retinal eccentricity. This placed the stimulus on the parafovea, where MPOD is negligible. Participants completed five trials at each locus. The two loci were compared to yield MPOD at 30-min of eccentricity. MPOD was measured at baseline, and after 4-, 8- and 12-month supplementation.

#### 2.3.2. Serum L + Z Levels

In addition to measuring CNS L + Z levels (as MPOD), L + Z were measured in the serum via high performance liquid chromatography (HPLC) to confirm participant compliance. The methods used to collect serum samples and measure L and Z concentration have been presented previously [25].

#### 2.3.3. Cognitive Function

Cognitive function was tested using a computerized test battery (CNS Vital Signs; Morrisville, NC, USA) at baseline, 4-, 8- and 12-month time points [26]. The procedure used to collect cognitive function data was presented previously [18]. Briefly, in order to complete computerized tests, participants were seated in front of a Dell E 2211H 18-inch monitor with a full QWERTY keyboard. A research assistant familiar with the test procedure remained in the room with the participant during the entire test protocol. Visual acuity of 20:40 or better was confirmed prior to enrollment. Prior to each test set, participants were given a practice session to confirm that test instructions were clear.

Individual functional tests and cognitive domains computed by raw scores on these tests are listed in Table 2, and have been presented previously [18]. During the test session, participants were given the same sequence of individual tests, each designed to measure some aspect of cognitive function. Raw scores on these individual tests were used to calculate cognitive domain scores, which were used in statistical analysis. For example, average numbers of taps executed by the right and left hands on the Finger Tapping Test were combined with the number of correct responses on the Symbol-Digit Coding task to compute the Psychomotor Speed domain score. Errors on the Stroop Task, Continuous Performance Task and Shifting Attention Task were combined to compute the Complex Attention domain score. For a complete list of tests administered, domains computed and computation parameters, see Table 2 and [18].

#### 2.3.4. Statistical Analyses and other Considerations

All statistical analyses were performed using SPSS version 23 (IBM) with *p* < 0.05 as the criterion for significance. The following domain scores were compared in statistical analyses: verbal memory, visual memory, reasoning, executive function, psychomotor speed, complex attention and cognitive flexibility. All tests were one-tailed, given the directional nature of the a priori hypotheses, specified below. The relationship between nutritional status and cognitive function was analyzed in two ways, as follows:

First, domain scores from participants on the placebo were directly compared against domain scores from participants taking the active supplement. To make these comparisons, difference scores were computed between baseline and 12-month time points for each cognitive domain tested, and those difference scores were compared between groups receiving placebo and those receiving the active supplement, for each domain. This comparison tests whether or not supplementation with L + Z was able to improve MPOD (the study biomarker of CNS xanthophyll levels) and whether such increases could affect cognitive function.

The second set of comparisons was aimed at addressing two major issues commonly seen in nutritional trials: poor compliance and dietary change. Non-compliance with the study regimen is seen in a number of clinical trials, from drug trials to nutrition trials, and we expected that some participants would not consistently take their nutritional supplement. Those in the active group who did not consistently take their supplements would, we predicted, have performance similar to placebo participants, rather than to other active supplement users.

The issue of dietary change is more complex. Although many nutrition trials (this trial included) are double-masked, to adhere to the tenets of the Declaration of Helsinki, participants should to the extent possible be informed about the nature of the trial. This model is most commonly used in drug trials, where participants either cannot acquire or would have some difficulty acquiring the active agent being tested. In nutrition trials that follow this “gold standard” randomized, double-masked, placebo-controlled trial model, once participants know the identity of the test supplement and the motivation for testing it, they can do individual research on the ingredients within that supplement and actually acquire the test molecules via the diet (or, if available, as is true in this case, in supplement form). In the current study, for example, participants were told at baseline that they would be taking either an active supplement containing L + Z or a visually identical placebo to examine putative benefits of these molecules on cognitive function. Given that fact, we expected that participants who researched the foods that naturally contain L and Z to learn more about the molecules might (even unintentionally) start increasing intakes of L- and Z-containing foods. For placebo group participants, we predicted that these dietary changes (or the frank obtaining of actives in the dietary supplement market) would cause performance to look more like the performance of active supplement users. Finally, it is well known that participants in clinical trials with diet questionnaires will unconsciously improve their diet (and in some cases other aspects of their lifestyle (exercise, alcohol intake and so forth) over the course of the study—perhaps simply because they know someone is “watching”.

In an attempt to determine whether or not participants changed dietary intakes, a food frequency questionnaire (FFQ) was collected at baseline and 12-month time points to measure fruit and vegetable intake. Participants in both groups tended to slightly increase the number of servings of fruits and vegetables consumed per day between baseline (3+ servings of fruits + vegetables per day) and 12 months (4+ servings/day). With respect to the compliance issue, compliance calls, pill counts and serum analysis suggested that 6 out of 37 participants in the active group were not fully compliant over the course of the year with the supplement regiment (these participants reported forgetting to take pills in at least three compliance phone calls), and thus would be less likely to show changes in MP optical density, the study biomarker of increased L + Z levels in the CNS. Finally, past research [27] has suggested that a small subset of the population is likely unable to absorb and/or appropriately traffic L and Z, and thus would not show serum and retinal responses even if they were fully compliant with the supplementation regimen.

To address these issues statistically, difference scores between MPOD at baseline and 12 months were computed. All participants who showed an increase of 0.10+ log units of MPOD, regardless of randomized group membership at study entry, were then grouped (*n* = 27) and were compared against participants whose MPOD did not increase (*n* = 24). Cognitive performance was then compared between those whose MPOD increased vs. those who did not increase. This comparison indicates whether or not increases in CNS L + Z (as predicted by MPOD measures) can improve cognitive function, irrespective of what caused the increased L + Z status.

## 3. Results

### 3.1. Retinal L + Z Levels

At baseline, MPOD in the study sample was higher (*M* = 0.48 ± 0.18) than previously published averages for healthy adults (*M* = approximately 0.3; [28,29]), indicating that the sample was relatively well-nourished. Following one year of supplementation, those on the active supplement increased significantly (*p* < 0.001) between the baseline (*M* = 0.47 ± 0.18) and 12-month (*M* = 0.56 ± 0.16) time-points. The participants on placebo did not increase significantly (baseline: 0.40 ± 0.12, 12-month: 0.44 ± 0.18; *p* > 0.05).

### 3.2. Serum L + Z Levels

**Figure 1:**
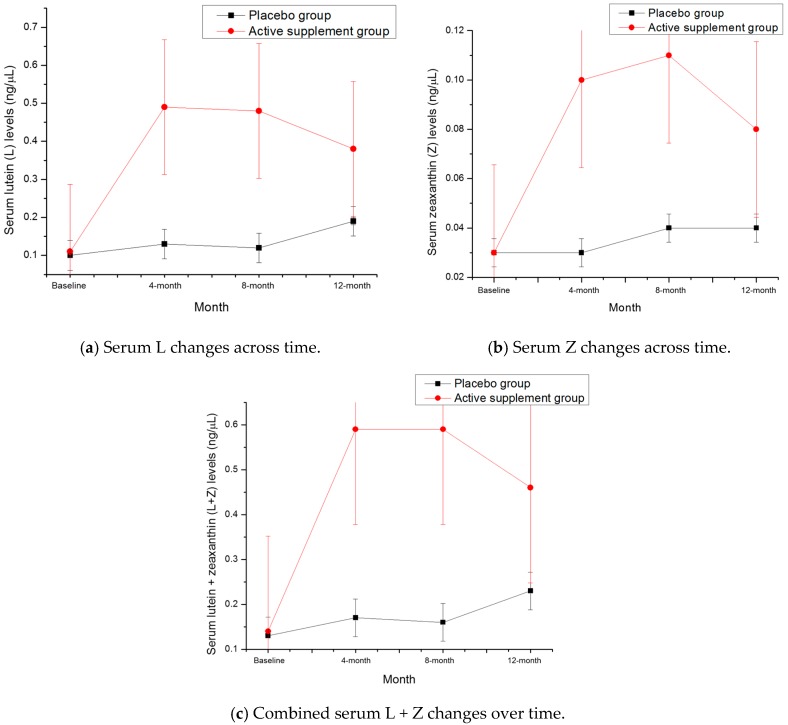
Serum changes from baseline to 12-month time points in: (**a**) lutein (L); (**b**) zeaxanthin (Z); and (**c**) and lutein + zeaxanthin (L + Z). Error bars represent the standard deviation of the mean at each time point.

At baseline, serum L, Z and L + Z levels were not significantly different between the participants randomized into the active supplement group vs. the placebo group (see Table 3). Following one year of supplementation, serum L (*p* = 0.029), Z (*p* = 0.011) and L + Z (*p* = 0.023) levels were all significantly higher in the active supplement group than the placebo group (see Table 4, Figure 1).

### 3.3. Cognitive Function

At baseline, participants were not significantly different from each other on any of the cognitive domains tested. Given the fact that participants were tested four times throughout the course of the study, practice effects were anticipated, and seen, regardless of group identity (see Table 5).

**Figure 2:**
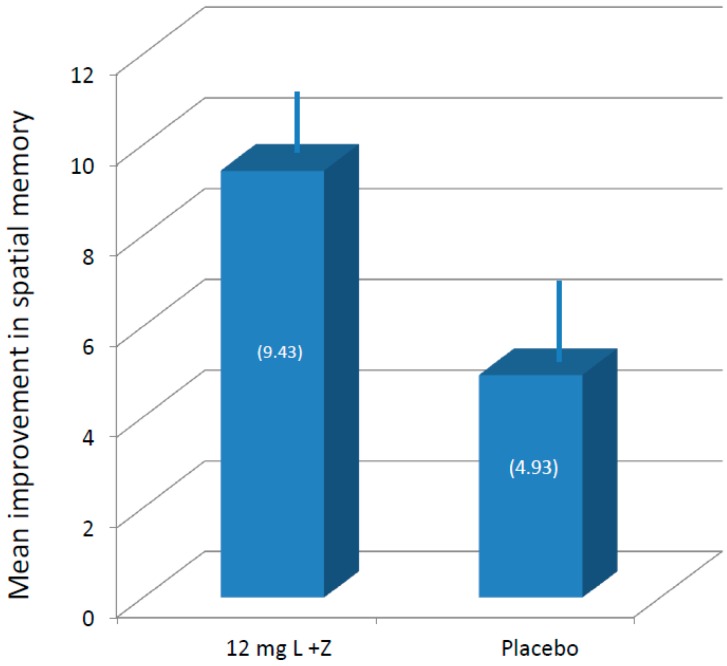
Average (with standard error of the mean, SEM) improvement in spatial memory following one-year supplementation. L, lutein; Z, zeaxanthin.

As mentioned previously, not all members of the supplement group were compliant with the intervention, and some of the members of the placebo group also had increases in MPOD (likely due to dietary changes since the intervention was an entire year). Consequently, analyses were conducted in two ways: by supplement group, and by percent increase in MPOD, regardless of group membership. With respect to supplement status, after 12 months of supplementation, participants taking the active supplement had significantly higher performance on visual memory tasks (*p* < 0.04) than those participants taking the placebo (see Figure 2). Analysis of the Reliable Change Index (RCI) using a standard criterion of 1.96 for both groups suggested that the effects seen in the active supplement group were not simply due to practice effects (RCI active = 6.77; RCI placebo = 1.88).

**Figure 3:**
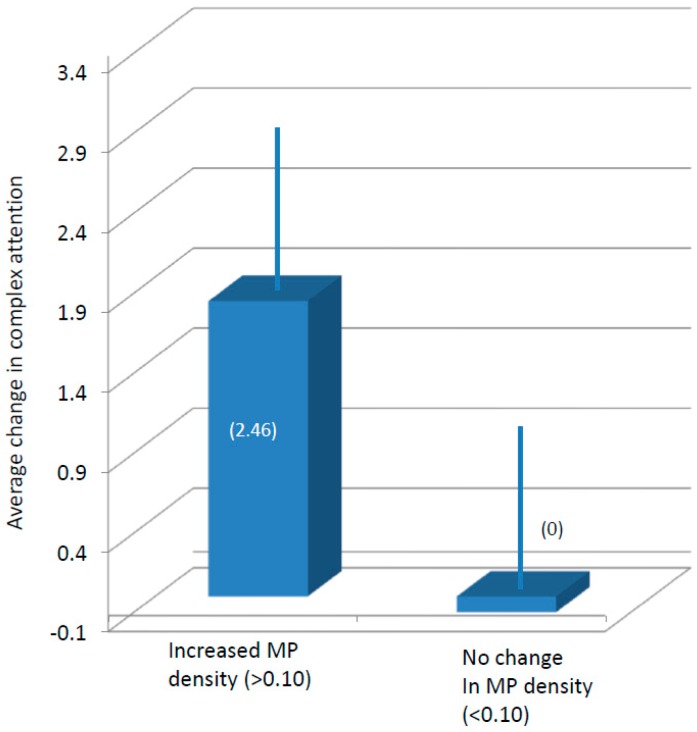
Average (and standard error of the mean (SEM)) change in complex attention segregated according to changes in macular pigment (MP) density.

**Figure 4:**
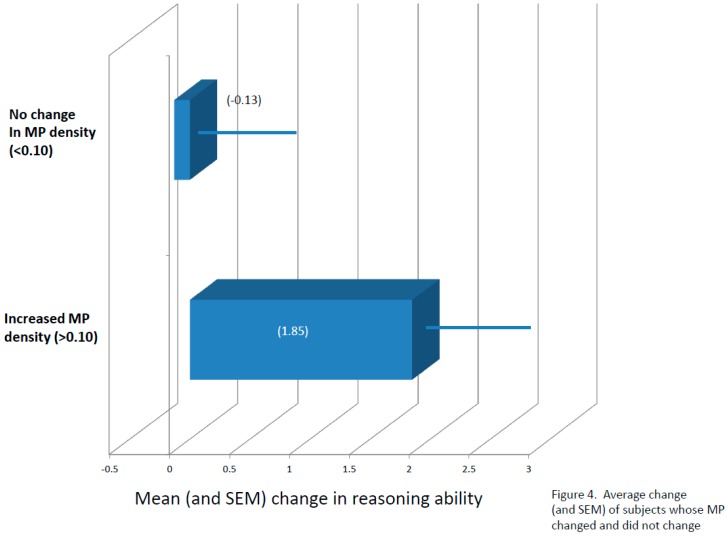
Average change (and SEM) of subjects whose MP changed and did not change.

With respect to percent increase in MPOD, baseline MPOD was subtracted from 12-month MPOD scores. Participants who had numerical increases of 0.10 or greater were classified as “increasers”, and participants who did not increase by a minimum of 0.10 were classified as “flat liners”, regardless of supplement group assignment. Compared to “flat liners”, participants with improvements of at least 0.10 for MPOD not only maintained significant differences in visual memory (*p* < 0.05) also scored significantly higher in the complex attention (*p* < 0.04) and reasoning ability (*p* < 0.05) tasks (see Figure 3 and Figure 4). Analysis of the RCIs for each task suggested, again, that the differences seen in complex attention (RCI for “increasers” = 2.02; RCI for “flat liners” = 0.00) and likely reasoning ability (RCI for “increasers” = 1.94; RCI for “flat liners” = 0.18) were not due simply to practice effects.

## 4. Discussion

This randomized, double-masked, placebo-controlled study tested the effects of one year of nutritional supplementation (10 mg of L and 2 mg of Z) on the cognitive function of healthy, well-nourished college students. L and Z were picked because they are commonly available in supplement form (often marketed for eye health) and the xanthophylls are commonly found in food (e.g., supplementing spinach will directly increase retinal levels [27]). They can also be easily measured in central nervous tissue (neural retina) [8] and are found in some key locations in the brain, such as hippocampus, and frontal and occipital cortex [9]. We originally speculated that changing cognitive function via a simple dietary intervention in young well-nourished college students was unlikely. Past studies, such as the companion older adult sample tested in the larger study from which these data were drawn [18], and others (e.g., [19]) have shown improvements in cognitive function in older adults. Older adult samples tend to have baseline cognitive function that is more variable (e.g., [10,11,13]), with a higher proportion of individuals who are at risk for dementia. Consequently, the older adult population is likely to an easier population in which to demonstrate change. This young adult, highly educated sample was selected deliberately to isolate treatment effects that were not likely to be confounded by education status, underlying nutritional deficiency, or progression toward cognitive impairment. Nonetheless, we were able to measure several distinct cognitive benefits that were directly tied to increasing central nervous system levels of L and Z, as shown by increased MPOD, even though the starting MPOD levels were substantially higher than those previously reported for the general population.

The most significant effect according to treatment was visual memory: the treated group changed but the placebos did not. In a recent study that tested retinal and brain L and Z levels in human decedents, L tended to be relatively highly concentrated in occipital cortex, and L + Z levels in neural retina tended to be highly (*r* = 0.78) correlated to brain levels of the macular carotenoids in occipital lobe [9]. This current study represents the first of its kind that suggests that increasing CNS L + Z can also improve performance in a function that is mediated in large part by occipital cortex, which preferentially accumulates L + Z relative to the other carotenoids more commonly consumed in the diet [30] in young, otherwise healthy, high performing subjects.

Visual memory is a relatively low-level cognitive function. Sometimes referred to as stimulus memory [31], it reflects dynamic cortical changes resulting from the storage or processing of visual stimuli. It is the stage that precedes processing by other structures such as the hippocampus which encodes more complex visual memories such as maps or scenes. Visual memory is hard to distinguish from attention since some aspects of the stimulus must be attended to in order to enter visual memory in the first place [32]. Characteristics such as visual memory, attention and processing speed have all, in turn, been related to improved reasoning ability [22,33].

In our study, the xanthophyll intervention was related to all three of these variables: visual memory, complex attention and reasoning ability. Specifically, improved visual memory was related to the treatment; complex attention and reasoning ability were improved only if significant increases in MP were also achieved. The fact that L + Z supplementation was related specifically to these variables in the younger adults may not be coincidence. As noted, these three cognitive functions are, in some ways, related to each other. Our results further suggest, however, that they are physiologically connected in a way that xanthophylls could improve, even in the young, who are, theoretically, also near ceiling in these functions. Earlier data from our lab has indicated that speed of processing in young subjects can also be changed by changing MPOD [14,15]. It is important to note that the intervention was not related to change in any of the other cognitive functions tested, and some of the functions that improved in the companion older adult sample [18] did not also improve in the young adult sample. Additional neuroimaging analyses of the entire study sample, including the older adult sample, suggests that L + Z supplementation improves white matter integrity in regions of brain known to show white matter declines in older age, and in those at risk for dementia [34]. Supplementation with L + Z also buffers against age-related declines in verbal learning and memory and improves cerebral perfusion in older adults [17]. Consequently, L + Z might be serving multiple functions in the CNS, some of which are more important in older age.

One common factor that could affect all of these functions is a general improvement in neural efficiency, a hypothesis proposed for L + Z in 2010 [35]. Hence, increasing central levels of L + Z could improve neural efficiency, which would both speed neural conduction while simultaneously reducing the likelihood of cross-talk between axons. Such basic effects can influence higher functions. The latter, for instance, could lower interference allowing multiple representations within the brain to be maintained and operated upon. We are currently using neuroimaging methods to assess these possibilities.

In the current study, several limitations should be considered with respect to generalizability. As mentioned previously, this sample was selected with the intention of avoiding both underlying nutritional deficiency that can contribute to poor cognitive performance, and cognitive differences between subjects that are due to differences in education level. Consequently, we ended up with a sample that should have been difficult to improve. The fact that these participants were well-nourished and cognitively high performing at baseline may limit generalizability to those with, for example, cognitive and neurological disease states.

## Figures and Tables

**Table 1:** Baseline characteristics of placebo participants vs. those on the active supplement.

Group	Age (Years)	Body Mass Index	Gender	Ethnicity	Race	Years of Education	MP Optical Density
Active, Mean ± SD	21.50 ± 2.69	24.15 ± 3.86	21 M, 16 F	*n* = 35 non-Hispanic *n* = 2 Hispanic	*n* = 30 White *n* = 4 Black *n* = 1 pan-Asian *n* = 2 Latino	12+	0.47 ± 0.18
Placebo, Mean ± SD	20.50 ± 1.91	23.03 ± 3.98	8 M, 6 F	*n* = 14 non-Hispanic	*n* = 10 White *n* = 2 Black *n* = 2 pan-Asian	12+	0.39 ± 0.12

None of the differences between the groups were statistically significant at baseline (*p* > 0.05). SD = standard deviation. M = male; F = female.

**Table 2:** Individual tests administered and cognitive domains evaluated in the test battery.

Domain	Corresponding Tests	Computation Procedure
Verbal Memory (VeM)	Verbal Memory Test	Correct hits for presented words + correct passes on distractors for tests immediately after presentation and after a 30-min delay.
Visual Memory (ViM)	Visual Memory Test	Correct hits for presented shapes and symbols + correct passes on distractors for tests immediately after presentation and after a 30-min delay.
Reasoning (R)	Non-Verbal Reasoning Test (NVRT)	Correct responses on the NVRT − commission errors on the NVRT.
Executive Function (EF)	Shifting Attention Test (SAT)	Correct responses on the SAT − errors on the SAT.
Psychomotor Speed (PmS)	Finger Tapping Test (FTT) Symbol-Digit Coding Test (SDC)	Average number of taps on the FTT with the right hand + average number of taps with the left hand + number of correct responses on the SDC
Complex Attention (CA)	Stroop Test (ST) SAT Continuous Performance Task (CPT)	Commission errors on the ST + Errors on the SAT + Commission and omission errors on the CPT
Cognitive Flexibility (CF)	SAT ST	Correct responses on the SAT − errors on the SAT − Commission errors on the ST

Note: Raw scores computed as described above were used in all statistical analyses.

**Table 3:** Baseline serum levels by supplement group.

Group	Serum Lutein (ng/μL)	Serum Zeaxanthin (ng/μL)	Lutein + Zeaxanthin (ng/μL)
Active, Mean ± SD	0.11 ± 0.07	0.03 ± 0.02	0.14 ± 0.08
Placebo, Mean ± SD	0.10 ± 0.03	0.03 ± 0.02	0.13 ± 0.04

None of the differences between the groups were statistically significant at baseline.

**Table 4:** Serum levels following one year of supplementation.

Group	Serum Lutein (ng/μL)	Serum Zeaxanthin (ng/μL)	Lutein + Zeaxanthin (ng/μL)
Active, Mean ± SD	0.38 ± 0.28	0.08 ± 0.05	0.46 ± 0.32
Placebo, Mean ± SD	0.19 ± 0.19	0.04 ± 0.04	0.23 ± 0.23

Note: Statistically significant differences were seen between the placebo group and the control group for serum L (*p* = 0.029), Z (*p* = 0.011), and L + Z (*p* = 0.023) following one year of supplementation.

**Table 5:** Practice effects demonstrated by improvement in cognitive function across domains, regardless of supplementation status.

Group	Verbal Memory	Visual Memory	Reasoning Ability	Executive Function	Complex Attention *	Cognitive Flexibility
Baseline Mean, all subjects	54.22	41.04	9.06	53.73	7.38	52.02
12-Month Mean, all subjects	56.26	49.24	10.20	62.59	5.98	60.65
Average change between baseline and 12 months, placebo group participants	3.57	4.93	1.64	8.86	0.46	8.43

* Lower score = fewer errors; for all other scores, an increase indicates improved performance.

## Registry Information

ClinicalTrials.gov number, NCT02023645.

## Abbreviations

L	lutein
Z	zeaxanthin
MPOD	macular pigment optical density
MZ	meso-zeaxanthin
CNS	central nervous system
MP	macular pigment
HPLC	high-performance liquid chromatography
FFQ	food frequency questionnaire
RCI	reliable change index
VeM	verbal memory
ViM	visual memory
R	reasoning
EF	executive function
PmS	psychomotor speed
CA	complex attention
CF	cognitive flexibility
NVRT	non-verbal reasoning test
SAT	shifting attention test
FTT	finger tapping test
SDC	symbol-digit coding
ST	Stroop test
CPT	continuous performance task
